# Utilization of artificial circular RNAs as miRNA sponges and anti-PD-1 scFv expression platforms to suppress hepatocellular carcinoma progression

**DOI:** 10.3389/fimmu.2025.1609165

**Published:** 2025-06-11

**Authors:** Yongping Lai, Fei Wang, Guang Cai, Yingying Li, Jiaxin Weng, Feifan Cai, Leijie Cai, Niangmei Cheng, Bixing Zhao, Yongyi Zeng

**Affiliations:** ^1^ Department of Hepatopancreatobiliary Surgery, First Affiliated Hospital of Fujian Medical University, Fuzhou, China; ^2^ Department of Hepatobiliary Pancreatic Surgery, Mengchao Hepatobiliary Hospital of Fujian Medical University, Fuzhou, Fujian, China; ^3^ The United Innovation of Mengchao Hepatobiliary Technology Key Laboratory of Fujian Province, Mengchao Hepatobiliary Hospital of Fujian Medical University, Fuzhou, China; ^4^ Mengchao Med-X Center, Fuzhou University, Fuzhou, China

**Keywords:** hepatocellular carcinoma, circular RNA, miRNA sponge, anti-PD-1, scFv antibody, immunotherapy

## Abstract

**Background:**

Hepatocellular carcinoma (HCC) is characterized by a complex interplay of genetic and epigenetic alterations that contribute to its aggressive nature and resistance to conventional therapies. The recent advent of immune checkpoint inhibitors has shown promise in enhancing the immune system's ability to target cancer cells. However, the efficacy of these therapies is often hindered by the tumor's immunosuppressive microenvironment. Circular RNAs (circRNAs), a class of non-coding RNAs, have emerged as promising candidates for the development of novel therapeutics due to their unique properties, including resistance to degradation and the ability to act as miRNA sponges.

**Methods:**

In this study, we engineered artificial circRNAs to target oncogenic miRNAs and to express anti-PD-1 scFv antibodies, aiming to simultaneously disrupt oncogenic pathways and enhance the immune response against HCC.

**Results:**

Our results demonstrate that the engineered circRNAs effectively sponge miR-25, leading to subsequent inhibition of HCC cell proliferation and angiogenesis. Moreover, the expression of anti-PD-1 scFv antibodies from the circRNAs significantly enhanced the cytotoxic T-cell response against HCC cells. *In vivo* studies revealed a significant reduction in tumor volume and prolonged survival in mice treated with the engineered circRNAs compared to controls.

**Conclusions:**

Our findings highlight the potential of artificial circRNAs as a novel therapeutic strategy for HCC. By harnessing their ability to act as miRNA sponges and to express immunomodulatory proteins, these engineered circRNAs offer a promising approach to overcome the challenges associated with HCC therapy.

## Introduction

Messenger RNA (mRNA) is widely recognized for its pivotal roles within biological systems. However, the brevity of its half-life imposes inherent limitations on its utility. Furthermore, the efficiency of mRNA translation is subject to constraints. In contrast, circular RNAs (circRNAs), which are abundant and stable RNA species in eukaryotic cells, are generated through a process known as back-splicing. A subset of endogenous circRNAs has been identified to harbor the capacity to encode proteins. Notably, Song et al. (1)reported that the circRNA ZKSCAN1 is capable of encoding a secretory peptide, circZKSaa, which impedes the progression of hepatocellular carcinoma (HCC) by facilitating the degradation of mechanistic target of rapamycin (mTOR). In another instance, circRNA circ-SMO was found to encode the SMO-193a.a peptide, a key component of the Hedgehog signaling pathway that fuels glioblastoma tumorigenesis and presents a promising therapeutic target for glioblastoma (2). The identification of circRNAs with protein-coding potential is an area of active research, and the prospect of employing circRNAs as vectors for protein expression is becoming increasingly feasible. Wesselhoeft et al. (3)engineered exogenous circular RNAs utilizing self-splicing introns, which achieved robust and sustained protein expression in eukaryotic cells. Chen et al. (4)significantly enhanced circRNA-mediated protein production by several orders of magnitude through the optimization of vector topology, 5’ and 3’ untranslated regions, internal ribosome entry sites, and the incorporation of synthetic aptamers that recruit the translation initiation machinery. In our prior studies, we harnessed circRNA to express tumor neoantigens, demonstrating that a circular RNA-based neoantigen vaccine is highly effective in tumor treatment and prevention across various murine tumor models, evoking a potent T-cell immune response (5).

In recent years, advancements in cancer immunotherapy have yielded significant clinical successes (6). Notably, monoclonal antibody-based immune checkpoint therapies have emerged as a potent strategy. These therapies leverage monoclonal antibodies to target and block inhibitory checkpoints on T cells, thereby circumventing the suppressive effects of tumor cells on T cell activity and enabling the selective elimination of tumor cells. The PD-1/PD-L1 axis has been a focal point of research, with numerous PD-1 monoclonal antibodies demonstrating notable therapeutic efficacy in clinical settings. Despite these advances, PD-1 monoclonal antibody therapy is not without limitations, including a relatively low response rate and the potential to trigger autoimmune reactions and other adverse effects. Consequently, there is a pressing clinical imperative to explore innovative antibody delivery systems and novel immunotherapeutic approaches.

Single-chain variable fragments (scFv) antibodies, which are constructed from the variable regions of heavy and light chains and linked by a short, flexible peptide, offer a promising alternative. These engineered antibodies maintain the specificity and affinity of their parent antibodies but exhibit several distinct advantages over their intact counterparts. The scFv antibodies are characterized by their reduced molecular size, enhanced tissue penetration, improved diffusion capabilities, diminished immunogenicity, shorter systemic retention, and simplified detection and purification processes. These attributes have positioned scFv antibodies as a subject of intense interest in the field of cancer immunotherapy. Rafiq et al. (7) have demonstrated the utility of scFv antibodies in augmenting the antitumor efficacy of CAR-T cells and bystander tumor-specific T cells by engineering these cells to secrete PD-1-blocking scFv. This strategy has shown promise in both syngeneic and xenogeneic mouse models of PD-L1+ hematological and solid tumors, underscoring the potential of scFv antibodies in enhancing immunotherapeutic outcomes.

MicroRNAs (miRNAs) are a class of endogenous small non-coding RNA molecules, composed of approximately 22 nucleotides. They are widely present in eukaryotes and play a crucial role in the regulation of gene expression (8). MiRNAs also exert significant effects in the initiation and progression of cancer, making them potential therapeutic targets. For instance, miR-25 has been shown to promote the growth, migration, and invasion of hepatocellular carcinoma cells (9, 10). Additionally, a study has demonstrated that cancer-derived exosomal miR-25-3p promotes pre-metastatic niche formation by inducing vascular permeability and angiogenesis, thereby facilitating liver metastasis in colorectal cancer (11).

Harnessing the miRNA sponge function of circular RNAs (circRNAs) to target and inhibit specific oncogenic miRNAs may serve as a strategy for cancer suppression. For example, Liu et al. designed an engineered circular RNA as a molecular sponge for miR-21, which inhibits the proliferation of gastric cancer cells (12).

In this study, we engineered a multifunctional circRNA that acts not only as a sponge for oncogenic miR-25 but also expresses anti-PD-1 single-chain variable fragment (scFv). This engineered circRNA demonstrated potent tumor-suppressive effects in both *in vitro* cellular assays and *in vivo* mouse models.

## Result

### Synthesis and validation of circular RNA silencing miR-25 and expressing α-PD1 scFV

**Figure 1:**
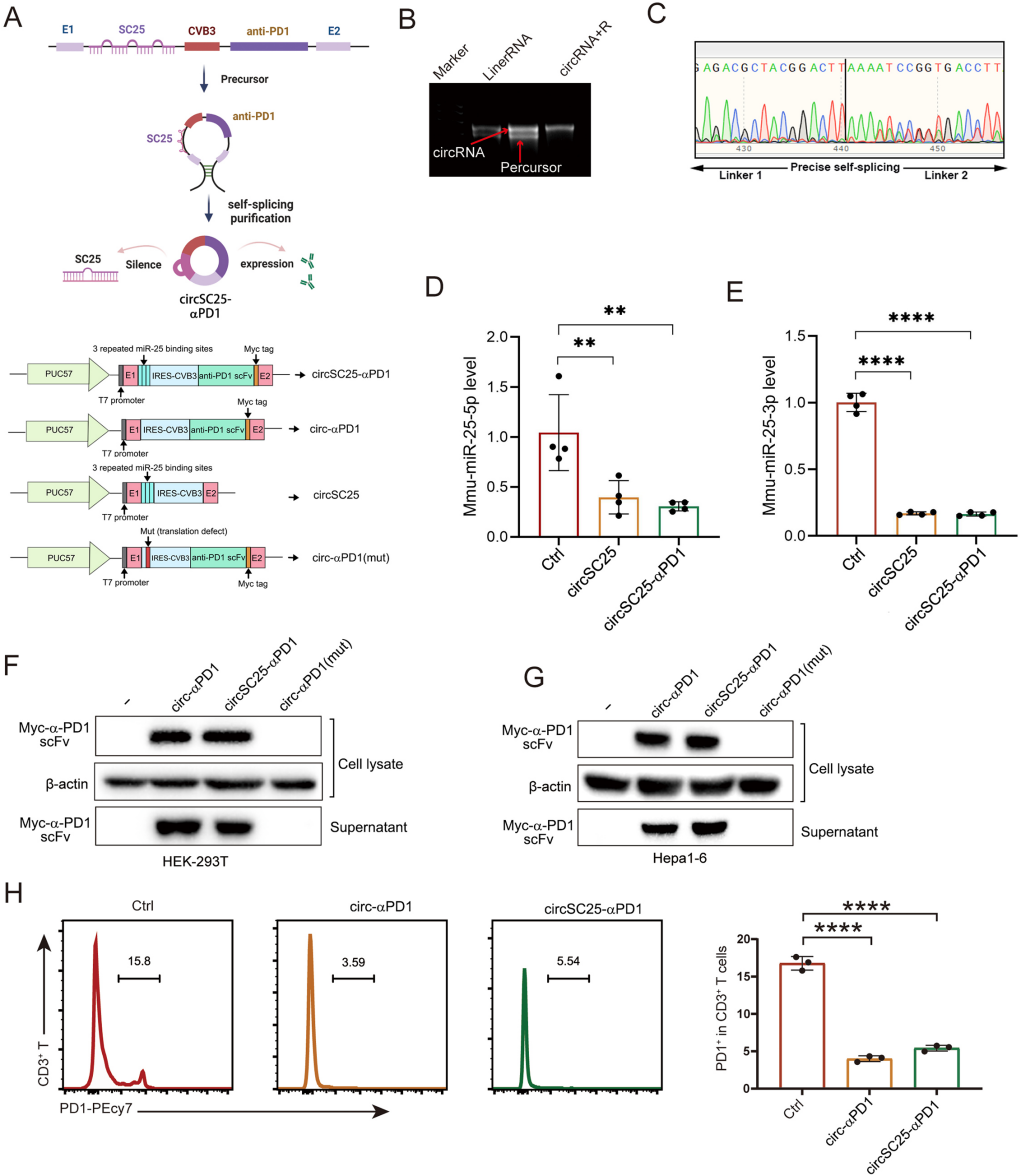
Synthesis and validation of circular RNA silencing miR-25 and expressing α-PD1 scFV. **(A)**Plasmid Sequence Design and Schematic Diagram of Engineered Circular RNA Synthesis. **(B)** Agarose gel confirmation of RNA circularization. **(C)** Sanger sequencing detection of circularization sites. **(D)** Western blot analysis showing the expression of α-PD1 in HEK293T and Hepa1–6 cells. **(F, G)** qPCR analysis demonstrating the effect of circRNA on miR-25 silencing. **(H)** Flow cytometry analysis of the blocking effect of circRNA on PD-1 expression in T cells. **, P < 0.01; ****, P < 0.0001.

To develop an engineered circular RNA (circRNA) capable of silencing miRNA-25 while simultaneously expressing α-PD1 single-chain variable fragment (scFV), we utilized the *in vitro* circularization method involving self-splicing introns. Based on previously published circularization methods (5), we constructed the circular RNA vector circSC25-αPD1, as well as control vector: Ctrl/circ-αPD1(mut) (with the IRES-CVB3 mutations, thereby abolishing its ability to drive the expression of downstream proteins (4), the synthesized circular RNA can also be called circ-αPD1(mut)), circSC25 (targeting miRNA-25 only), and circ-αPD1 (expressing α-PD1 scFV only). The sequence structures of different plasmids are shown in **Figure 1A**. RNA electrophoresis confirmed the formation of circRNA following the splicing reaction (**Figure 1B**). The presence of the circularization site was further verified by Sanger sequencing (**Figure 1C**). Subsequently, we transfected HEK-293T and Hepa1–6 cells with the circularized RNA to evaluate their silencing effect on miRNA-25 and expression efficiency of α-PD1 scFV. Quantitative PCR (qPCR) analysis demonstrated that the engineered circRNA effectively silenced both miRNA-25-3p (**Figure 1D**) and miRNA-25-5p (**Figure 1E**). Western blotting revealed that the circRNA efficiently expressed and secreted α-PD1 scFV extracellularly (**Figures 1F, G**). To assess the functionality of the expressed α-PD1 scFV, T cells were isolated from mouse spleens and activated by co-stimulation with CD3 and CD8 antibodies. The activated T cells were then co-cultured with cells transfected with the engineered circRNA. Flow cytometry analysis showed that the α-PD1 scFV effectively bound to PD1 on the surface of T cells (**Figures 1H, I**). Collectively, these results confirm that we have successfully constructed an engineered circRNA that can both silence miRNA-25 and express functional α-PD1 scFV. Future studies will focus on further investigating the functions of this engineered circRNA in HCC cells.

### Synthetic circRNA as a miR-25 sponge affects invasion, metastasis and proliferation of HCC cells

**Figure 2:**
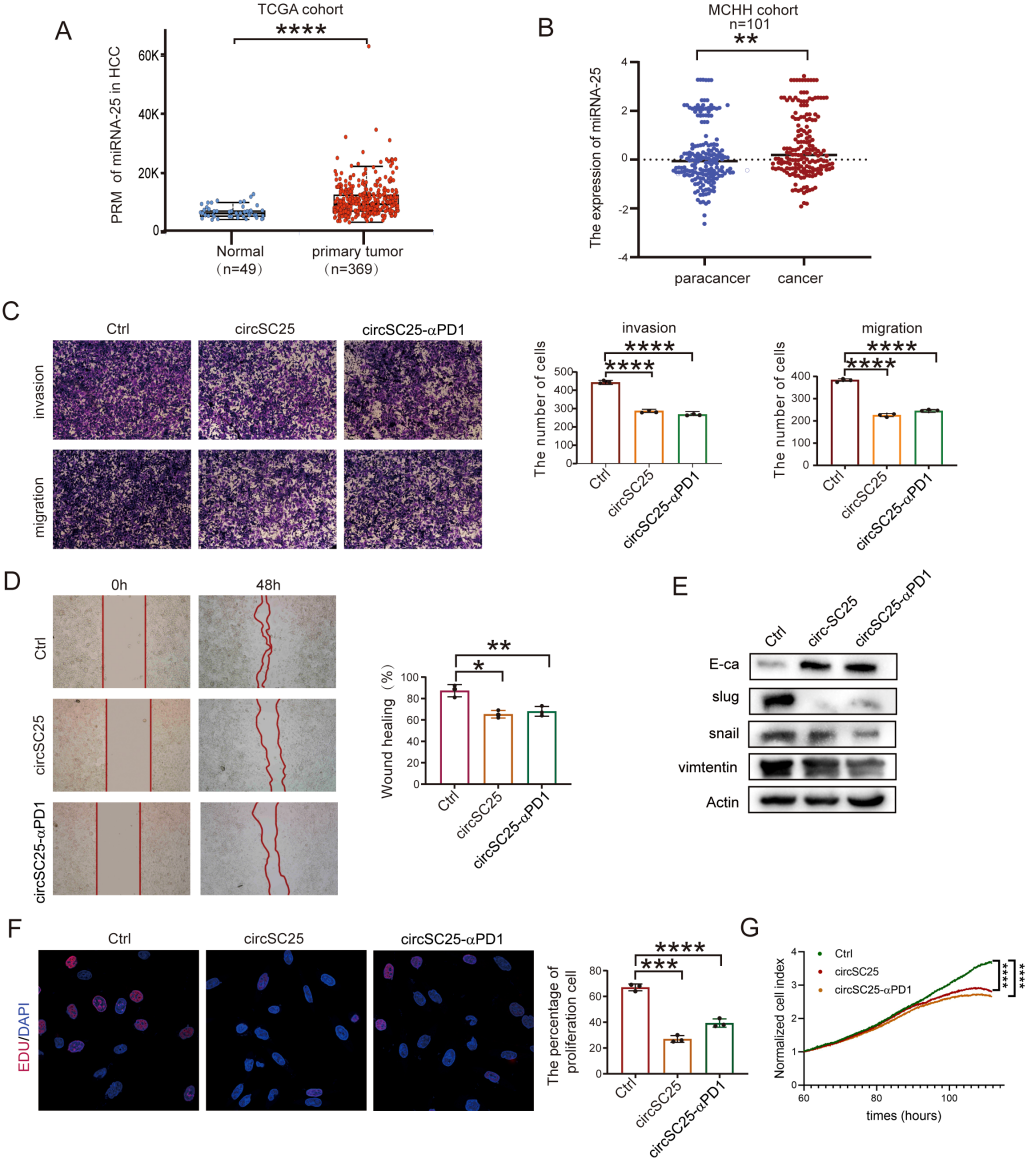
Engineered circRNA as a miR-25 sponge inhibits invasion, migration, and proliferation in HCC. **(A)** Relative expression levels of miR-25 in normal tissues (n = 49) and HCC tumor tissues (n = 369) from the TCGA cohort. **(B)** Expression of miR-25 detected by RT-qPCR in 101 HCC patients with paired tumor and para-tumor tissues. **(C)** Representative results and quantitative analysis of transwell cell migration and invasion assays after silencing miR-25 in Hepa1–6 cells. **(D)** Wound healing assays validate the effects of silencing miR-25 in Hepa1–6 Cells. **(E)** Western blot detection of epithelial (E-cadherin), mesenchymal (Vimentin), and EMT related transcription factors (Slug and Snail) in Hepa1–6 Cells with Silencing of miR-25. **(F)** EdU assays detect cell proliferation after silencing miR-25 in Hepa1–6 cells. **(G)** Real-time cell analysis (RTCA) assays validate cell proliferation after silencing miR-25 in Hepa1–6 cells. *, P < 0.05; **, P < 0.01; ***, P < 0.001; ****, P < 0.0001.

Based on microRNA sequence data from 369 HCC patients and 49 normal samples in the Cancer Genome Atlas (TCGA), we found that the expression of miRNA-25 was upregulated in HCC compared to normal samples (**Figure 2A**). To further verify the expression of miR-25 in HCC, RNA was extracted from tumor and adjacent non-tumor tissues of 101 HCC patients, and the expression of miR-25 in clinical samples was examined via quantitative PCR (qPCR) experiments. The results confirmed that miR-25 was significantly upregulated in HCC tissues compared to adjacent non-tumor tissues (**Figure 2B**).

To investigate the effect of miR-25 silencing on the malignant phenotype of HCC cells, we transfected the synthetic circRNA (circSC25 and circSC25-αPD1) into Hepa1–6 cells. Transwell experiments showed that miR-25 silencing significantly reduced the metastatic and invasive abilities of hepa1–6 cells (**Figure 2C**). This finding was further confirmed by wound healing assay, which demonstrated that miR-25 silencing impaired the migratory capacity of hepa1–6 cells (**Figure 2D**).

Subsequently, we further investigated the impact of miR-25 silencing on epithelial-mesenchymal transition (EMT) of HCC cells. Western blot analysis revealed that circSC25 and circSC25-αPD1 significantly upregulated the expression of the epithelial marker protein E-cadherin, while downregulating the mesenchymal marker protein vimentin. Additionally, the expression of EMT-related transcription factors Slug and Snail was also markedly reduced. These findings indicate that the synthetic circRNA significantly inhibits the EMT process in HCC cells (**Figure 2E**). Moreover, the results from the 5-ethynyl-2′-deoxyuridine (EdU) cell proliferation assay and real-time cellular analysis (RTCA) demonstrated that silencing miR-25 effectively inhibits the proliferation of HCC cells (**Figures 2F, G**).

### Synthetic circRNA targets miR-25 to inhibit angiogenesis

**Figure 3:**
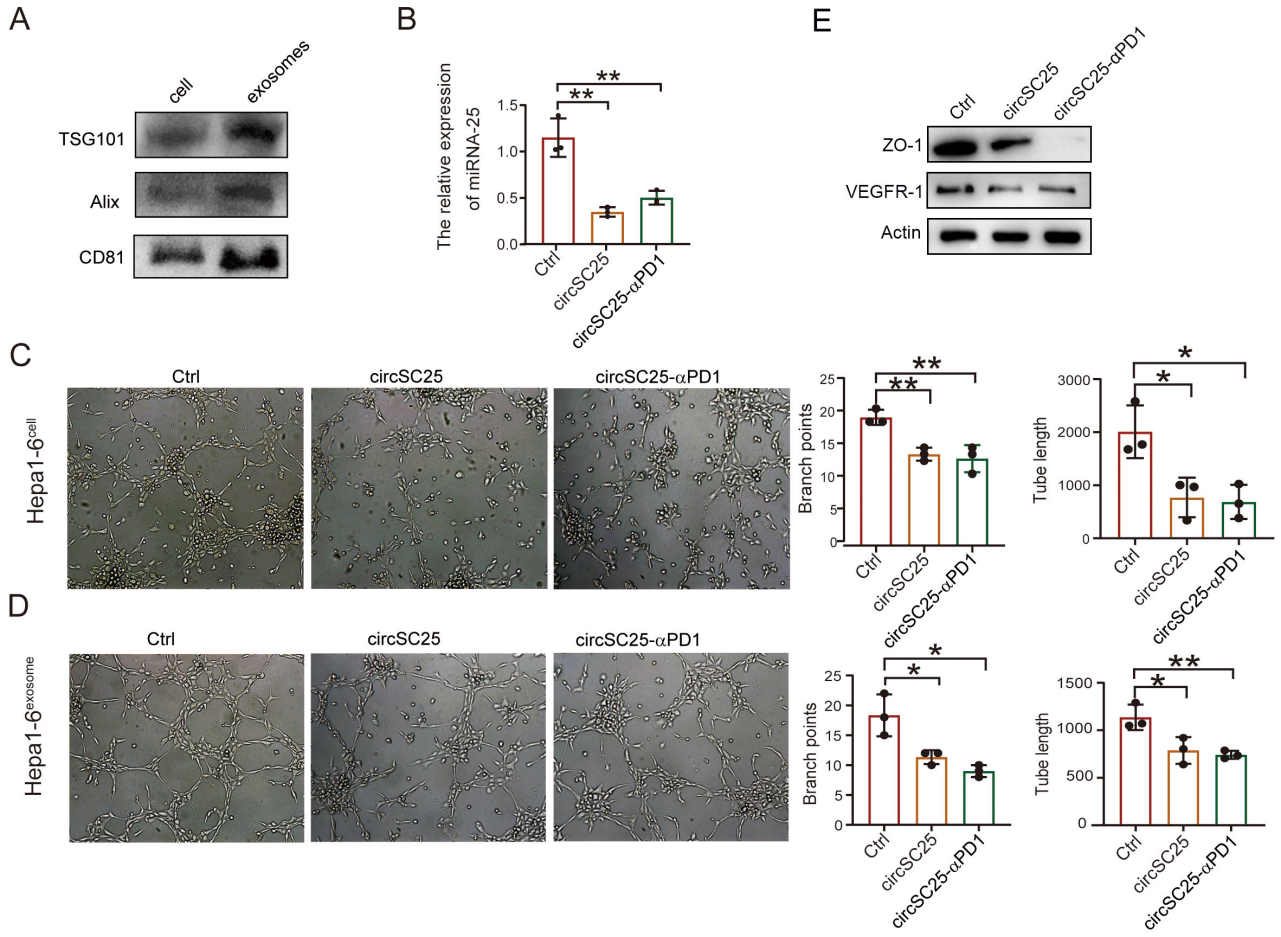
Engineered circRNA affects angiogenesis in liver cancer cells. **(A)** Western Blot detection of exosomal markers. **(B)** qPCR test confirm the silencing efficiency of miRNA-25 in exosomes. **(C)** The effect of heap1–6 cells transfected with engineered circRNA on angiogenesis. **(D)** The effect of exosomes secreted by heap1–6 cells transfected with engineered circRNA on angiogenesis. **(E)** Western blotting detection of ZO-1, VEGFR in HUVEC cells with co-culture with transfected heap1–6 cells. *, P < 0.05; **, P < 0.01.

Cancer-derived exosome miR-25-3p is known to promote liver metastasis of cancer by inducing vascular permeability and angiogenesis to promote the formation of a premetastatic niche (11). Therefore, we further explored the impact of silencing miR-25 using synthetic circRNA on tumor angiogenesis. We first isolated exosomes from the supernatant of Hepa1–6 cells transfected with synthetic circRNA. Western blot analysis of exosomal marker proteins confirmed the successful extraction of exosomes (**Figure 3A**). Detection of miR-25 expression in the exosomes revealed that synthetic circRNA reduced the levels of miR-25 in exosomes (**Figure 3B**). Results from the HUVEC capillary tube formation assay showed that synthetic circRNA transfection inhibited *in vitro* HUVEC capillary tube formation, regardless of whether the HUVECs were co-cultured with Hepa1–6 cells or treated with Hepa1–6 cell-derived exosomes (**Figures 3C, D**). Western blot analysis of angiogenesis-related proteins indicated that silencing miR-25 in HCC cells significantly upregulated the expression of VEGFR and tight junction protein ZO-1 (**Figure 3E**). Collectively, these results demonstrate that synthetic circRNA inhibits *in vitro* tumor angiogenesis by suppressing miR-25 expression in HCC cells.

### Therapeutic effect of artificial circRNA in subcutaneous tumor mouse model

**Figure 4:**
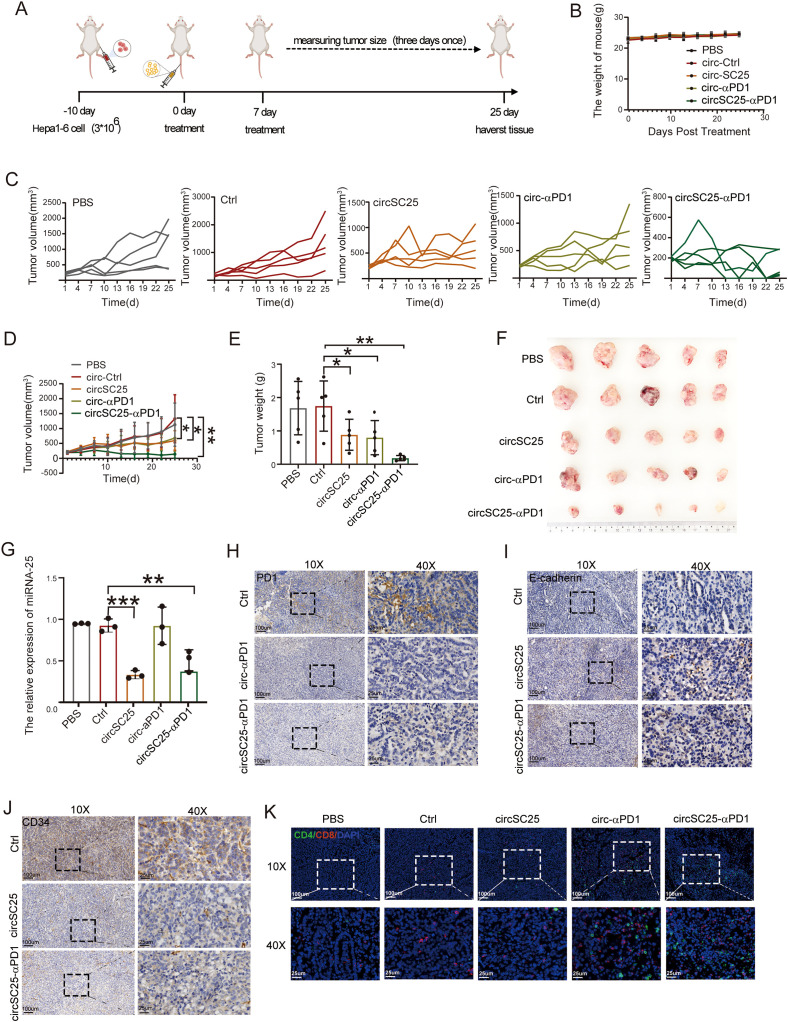
Anti-tumor efficacy of circRNAs in subcutaneous HCC model. **(A)** Treatment timeline of the experiment to evaluate the anti-tumor efficacy. **(B)** Body weight curves of mice during treatment. **(C)** Tumor growth curves of each group. **(D)** Average tumor growth curve of mice after treat with PBS, Ctrl, circSC25, circ-αPD1, circSC25-αPD1 (n = 5). **(E)** Tumor weight of each group. **(F)** Images of tumor size. **(G)** The silencing effect of miR-25 in tumor tissues. **(H-J)** Validation of PD-1, E-cadherin (E-Ca), and CD34 expression in tumor tissues by immunohistochemistry, Scale bars: 25 and 100 µm. **(K)** The representative immunofluorescence image of CD4^+^ and CD8^+^ T-cell infiltration in tumor tissues. Scale bars: 25 and 100 µm. *p < 0.05, **p < 0.01, ***p < 0.001.

To further validate the anti-tumor effects of synthetic circRNA *in vivo*, we established a subcutaneous Hepa1–6 tumor model in mice. Tumor-bearing mice were intravenously injected with LNP-circRNA-SC25-anti-PD1 via the tail vein on days 0 and 7 (**Figure 4A**). As shown in **Figure 4B**, neither the PBS control group nor the circRNA injection group exhibited significant changes in body weight, indicating that the treatments were well-tolerated by the mice. The anti-tumor efficacy of synthetic circRNA was evaluated by measuring tumor growth. Compared to the PBS and empty vector controls, silencing miR-25 alone or expressing anti-PD-1 scFv alone significantly inhibited tumor growth. As expected, the combination of miR-25 silencing and anti-PD-1 scFv expression in circRNA demonstrated the most pronounced tumor suppression (**Figures 4C-F**). To verify the silencing efficiency of miR-25, we extracted RNA from tumor tissues after treatment and measured miR-25 expression levels using qPCR. The results confirmed that circSC25 effectively reduced miR-25 expression in tumor tissues (**Figure 4G**). We further performed immunohistochemical staining on tumor tissues to assess the expression levels of PD-1, E-cadherin, and CD34. The results showed that silencing miR-25 significantly upregulated E-cadherin expression while downregulating CD34 expression in tumor tissues (**Figures 4I, J**). These findings validate the inhibitory effects of miR-25 silencing on EMT and tumor angiogenesis. Additionally, the expression of anti-PD-1 scFv significantly reduced PD-1 expression in tumor tissues (**Figure 4H**) and enhanced the infiltration of CD4^+^ T cells and CD8^+^ T cells into the tumor microenvironment (**Figure 4K**).

Collectively, these results demonstrate that the engineered synthetic circRNA effectively inhibits tumor growth, angiogenesis, EMT, and enhancing T cell infiltration in a mouse subcutaneous tumor model.

### Safety detection of artificial circRNA *in vivo*

**Figure 5:**
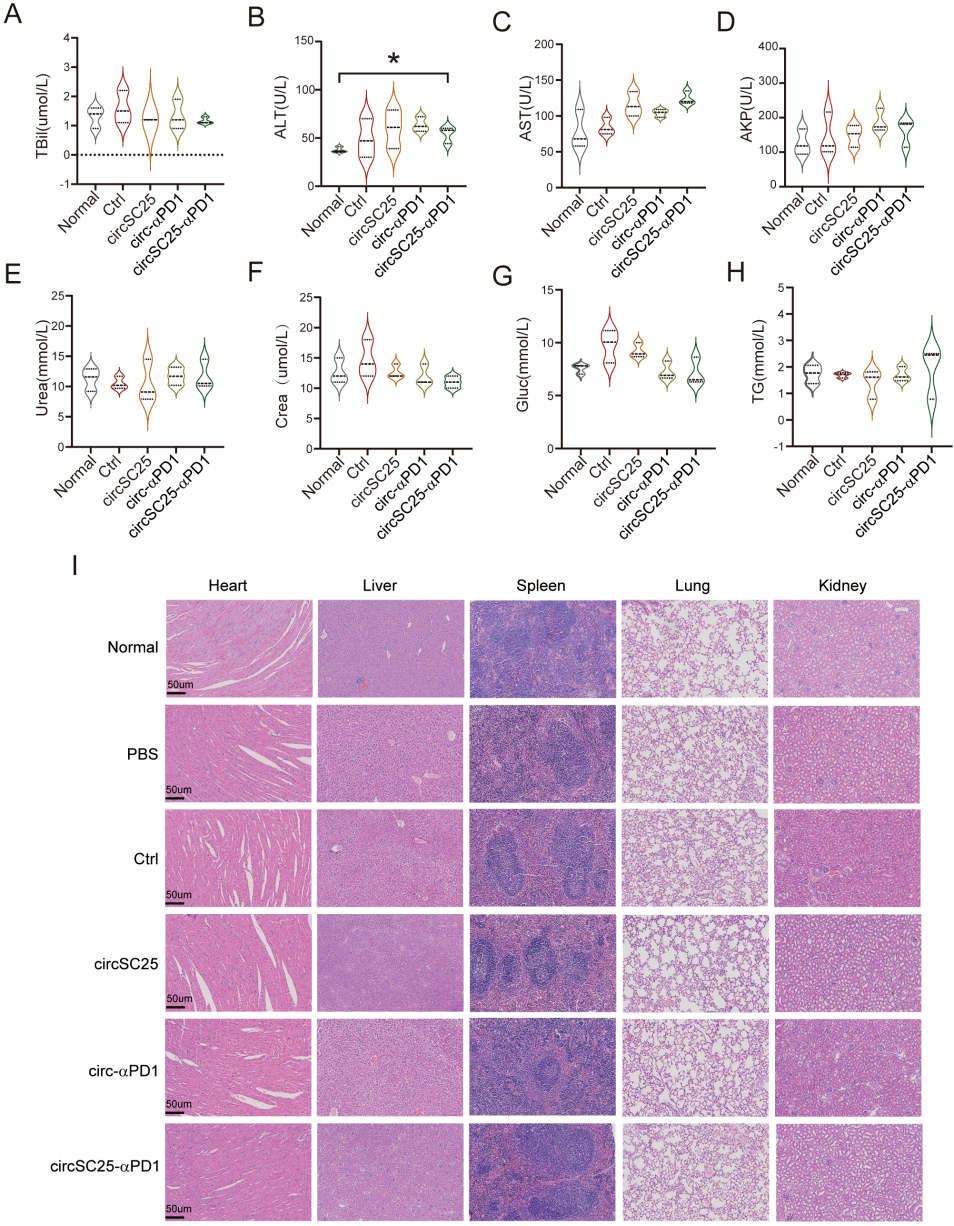
Safety detection of artificial circular RNA. **(A-H)** Biochemical indicators of peripheral blood serum Triglyceride (TG), Glucose (Glu), Serum Creatinine (Crea), Urea, Alk aline phosphatase (AKP), Aspartateamino transferase (AST), Alanineamino transferase (ALT), Total bilirubin (TBil) after treatment with circRNAs; **(B)** Assessment of H&E staining of major organ pathology in HCC subcutaneous tumor-bearing mice after treatment with circRNAs. *p<0.05. **(I)** Indicate that the scale bar of the image is 50 µm.

To evaluate the safety of the artificial circRNA *in vivo*, we harvested the major organs and plasma from mice in different treatment groups and used normal mice as controls. We assessed mouse plasma liver function indicators and found that the circRNA vaccine had no significant impact on liver function (**Figures 5A–H**). Systemic toxicity was evaluated by hematoxylin and eosin (H&E) staining of the major organs, and no significant toxic side effects were observed (**Figure 5I**).

### Tumor therapeutic efficacy of artificial circRNA in orthotopic HCC models

**Figure 6:**
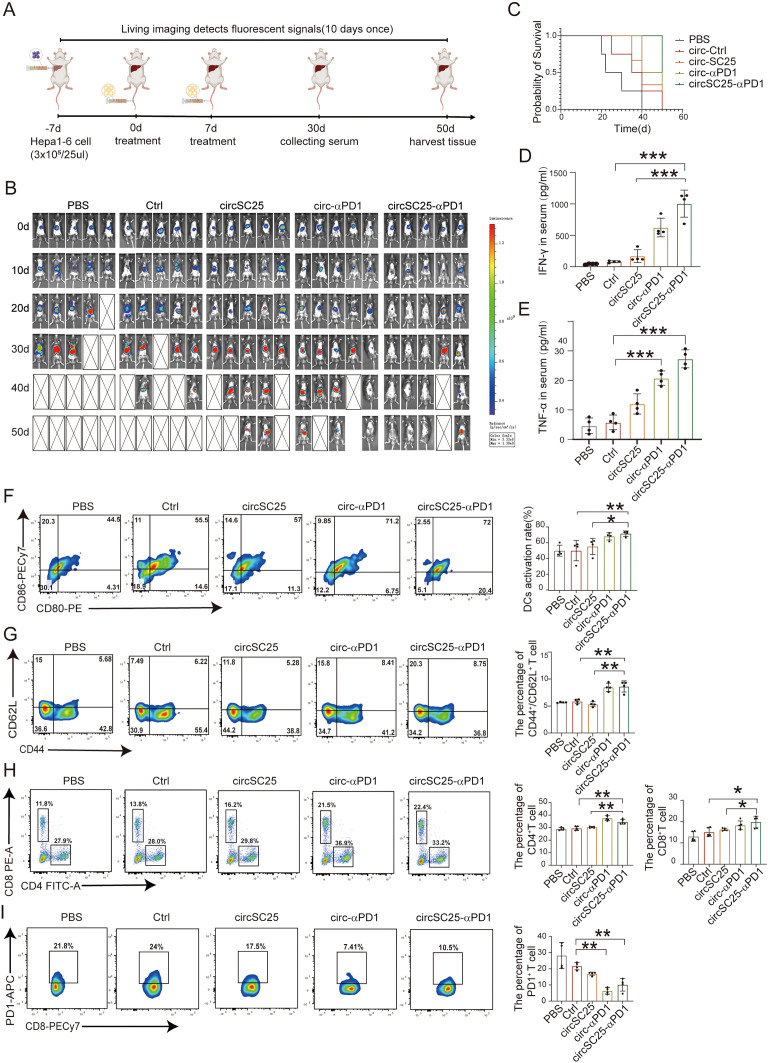
Anti-tumor efficacy of circRNAs in orthotopic HCC model. **(A)** Treatment timeline of the experiment to evaluate the anti-tumor efficacy. **(B)** Kaplan–Meier survival curves of PBS, Ctrl, circSC25, circ-αPD1, circSC25-αPD1. **(C)** Tumor burden monitoring of PBS, Ctrl, circSC25, circ-αPD1, circSC25-αPD1 treated mice by bioluminescence imaging (n=5). **(D, E)** Serum cytokine interferon-γ and tumor necrosis factor-alpha (TNF-α) release after circRNAs administration via ELISA assays. **(F)** Flow cytometry analysis the percentage of matured dendritic cells (DCs) in lymph nodes after different treatment. **(G)** Flow cytometry analysis the percentage of central memory T cells in spleen after different treatment and the statistical analysis. **(H)** Flow cytometry analysis the percentage of CD8^+^ and CD4^+^ T cells in spleen after different treatment and the statistical analysis. **(I)** Flow cytometry analysis the PD1 expression level in CD8^+^ T cells in spleen after different treatment and the statistical analysis. *p < 0.05, **p < 0.01, ***p < 0.001.

To further elucidate the anti-tumor effects of engineered circRNA, we established orthotopic Hepa1–6 HCC models in mice and treated tumor-bearing mice with two intravenous injections of LNP-circRNA via the tail vein (**Figure 6A**). Tumor progression was monitored every 10 days using IVIS bioluminescence imaging. As shown in **Figure 6B**, both circSC25 and circ-αPD1 treatments significantly inhibited tumor progression. Notably, treatment with circSC25-αPD1 exhibited the most pronounced anti-tumor effect, with 60% (3/5) of tumors being completely eradicated. Moreover, the overall survival time of mice in the circSC25-αPD1 group was significantly prolonged (**Figure 6C**). Then, peripheral blood samples were collected for ELISA to measure pro-inflammatory cytokines. The levels of IFN-γ and TNF-α in the serum of the combination therapy group were significantly higher than those in the control group (**Figures 6D, E**), indicating a more robust immune response in the combination therapy group.

Subsequently, we evaluated the effects on DC activation and T cell immunity. The activation status of mouse DCs was preliminarily assessed. The proportion of mature DCs in the anti-PD1 scFv group was higher compared to the PBS (p<0.001) and Ctrl (p<0.05) groups (**Figure 6G**). We then analyzed the effector T cells in the spleens of mice receiving various treatments using flow cytometry. The results showed that compared with the PBS (p<0.05) and Ctrl (p<0.05) groups, the percentage of CD4^+^ T cells and CD8+ T cells increased in the anti-PD1 scFv group (p<0.05) (**Figure 6H**). Additionally, the proportion of CD4^+^/CD62L^+^ T cells was significantly higher in the anti-PD1 scFv group than in the control group (p<0.05) (**Figure 6I**), indicating a more effective immune response. Flow cytometry was also used to analyze the expression level of PD1 on CD8^+^ T cells, and the results showed that anti-PD1 scFv expression effectively inhibited PD1 on the surface of CD8^+^ T cells (**Figure 6J**).

Therefore, our findings demonstrate that artificial circular RNA targeting oncogenic miRNA and expressing anti-PD-1 scFv antibody also exerts anti-tumor effects in an orthotopic HCC mouse tumor model.

## Discussion

MicroRNAs (miRNAs), a class of non-coding RNA molecules, play a significant role in the development of cancer. Extensive research on miRNA functions has confirmed that miRNAs are often deregulated in many types of cancer, where they can act as tumor suppressors or oncomiRs (13). For instance, the miR-200 family is downregulated in cancer and acts as a tumor suppressor involved in tumor metastasis and epithelial-mesenchymal transition (EMT) (14–16). On the other hand, miR-21 is significantly upregulated in tumors, serves as an oncomiR and promotes cell proliferation, metastasis, and drug resistance (17–19). Therefore, targeting miRNAs is an intriguing antitumor strategy, with miRNA mimics and molecules targeting miRNAs showing promise in preclinical studies, and some have advanced to clinical trial stages. For example, a liposomal miR-34a mimic (20) and a miR-16-based mimic microRNA (21) are under investigation. Additionally, utilizing artificial miRNA sponges to incapacitate oncomiRs represents a potential anticancer strategy. For instance, miR-23b in glioblastoma was inhibited with a miRNA sponge delivered via a lentiviral vector, demonstrating that the inhibition of this miRNA in glioma resulted in reduced tumor malignancy, suggesting its potential as an adjuvant anticancer therapy (22). Another study employed circular RNA (circRNA) as a molecular sponge for miR-21, showing significant suppression of downstream protein targets of miR-21 and inhibition of tumor cell growth *in vitro* (12). Our research also employs circRNA as a molecular sponge for miRNAs, and more importantly, our engineered circRNA not only functions as a sponge for miR-25 but also expresses the α-PD1 scFV, exerting a synergistic therapeutic effect on hepatocellular carcinoma by targeting the suppression of miR-25 and simultaneously functioning as an immune checkpoint inhibitor.

The expression levels of microRNAs (miRNAs) frequently undergo dysregulation in cancers, leading to their classification as either oncogenic miRNAs or tumor suppressor miRNAs. These molecules play a critical role in modulating key biological processes in cancer cells, including proliferation, apoptosis, invasion, metastasis, and drug resistance (23). Given their central role in the pathogenesis and progression of cancers, miRNAs have emerged as promising therapeutic targets. Targeted inhibition of oncogenic miRNAs and the use of miRNA mimics to bolster the activity of tumor suppressor are among the strategies that have garnered significant research interest. MiR-25, in particular, has been found to be significantly overexpressed in clinical HCC tissues relative to normal liver tissues, and its upregulation is correlated with adverse prognostic outcomes (24). Functionally, miR-25 has been implicated in promoting the growth, migration, and invasion of HCC cells (9, 10). Furthermore, emerging evidence suggests that exosomal miR-25-3p contributes to the formation of pre-metastatic niches and stimulates angiogenesis, thereby facilitating hepatic metastasis in colorectal cancer (11). Based on these findings, we propose that the targeted inhibition of miR-25 may have a multifaceted impact on the biological behavior of HCC cells. Our experimental results substantiate this hypothesis, demonstrating that a circRNA based miR-25 sponge, significantly curtails the proliferation, invasion, migration, and angiogenesis capabilities of HCC cells. Previous studies have also demonstrated that miR-25 targets multiple downstream genes to regulate EMT and metastasis in tumors, including ZEB2 (25), RhoGDI1 (9), PTEN (26), and FOXP2 (27). Additionally, exosomal miR-25 regulates angiogenesis by targeting KLF2 and KLF4 (11). Therefore, the engineered circRNA we designed, by sequestering miR-25, may affect the aforementioned multiple targets and ultimately inhibit EMT and angiogenesis in hepatocellular carcinoma. This approach offers a novel therapeutic avenue for the management of HCC and warrants further investigation into its potential clinical applications.

The molecular weight of single-chain variable fragments (scFvs) is approximately 25 kDa, which is considerably lower than that of full-length monoclonal antibodies (mAbs) at about 150 kDa. This reduced size confers several advantages to scFvs, including enhanced penetration into tumor tissues and more rapid clearance from the bloodstream, which may mitigate systemic side effects. Additionally, the lack of an Fc region in scFvs diminishes the risk of non-specific binding and potential adverse immune reactions (28). In a recent study, PD-1-TREM2 scFv-secreting chimeric antigen receptor T (CAR-T) cells exhibited potent tumor elimination in a subcutaneous colorectal cancer (CRC) mouse model (29). Furthermore, the targeted delivery of a PD-1-blocking scFv via CAR-T cells was shown to augment the antitumor activity of both the CAR-T cells and the bystander tumor-specific T cells (7). Fei et al. engineered an oncolytic herpes simplex virus vector expressing a human PD-1 single-chain variable fragment (hPD-1scFv), termed YST-OVH, which substantially amplified the activity of CD8^+^ T cells within mouse tumors and demonstrated significant antitumor effects across various humanized PD-1 mouse tumor models (30). In our study, we harnessed engineered circular RNA (circRNA) to express an anti-PD-1 scFv. This innovative approach not only markedly suppressed the growth of both subcutaneous and orthotopic tumors in mice but also potentiated the activation of dendritic cells (DCs) in the lymph nodes and facilitated T-cell infiltration at the tumor site, thereby enhancing the overall antitumor immune response.

**Figure 7:**
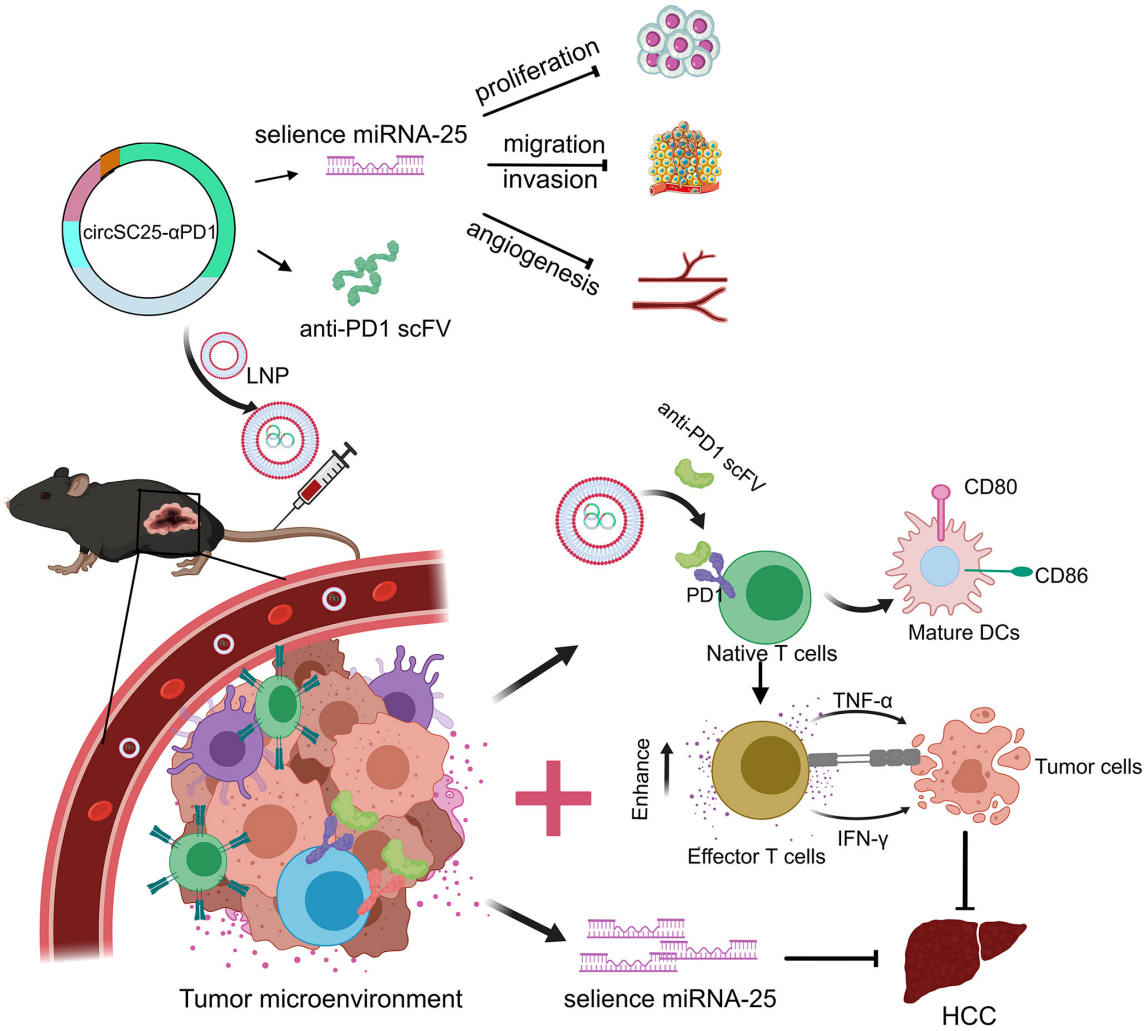
Schematic diagram of the engineered circRNA serving as a molecular sponge for miR-25 and expressing anti-PD1 scFv to synergistically inhibit hepatocellular carcinoma.

In conclusion, our study has engineered a multifunctional artificial circular RNA (circRNA) that serves dual roles: as a molecular sponge for miR-25 and as a vehicle for the expression of an anti-PD-1 scFv. This engineered circRNA mitigates tumor growth by targeting and inhibiting the oncogenic miR-25 in HCC cells, while concurrently blocking the PD-1/PD-L1 signaling within the tumor microenvironment, thereby exerting a synergistic antitumor effect (**Figure 7**). Our research provides experimental evidence for the potential of synthetic circRNAs as innovative tools in anticancer research and as candidates for future molecular therapeutic strategies.

## Materials and methods

### Plasmid construction and production of RNA

The plasmid structure comprises a circular RNA main body, homology arm, type I intron sequence, miR-25 silent sequence, IRES sequence, anti-PD-1 sequence, and linker sequence, which are cloned into the pUC57 vector. The specific plasmid sequence is detailed in **Supplementary Table S1**. To amplify the plasmid, it is first cloned into the pUC57 vector, and then the plasmid is amplified to produce a larger quantity. Subsequently, the plasmid is digested with endonuclease, and the circular skeleton region is isolated via nucleic acid electrophoresis. Circularization is then performed as previously described (5). Specifically, the circularized domain is transcribed using a T7 kit. The transcription product is treated with DNase I at 37°C for 20 minutes to eliminate uncyclized DNA, and the digested product is purified using column A. The quality and concentration of the purified product are then assessed. For the circularization step, 10 mM GTP is added to a 50 μL reaction system, and the mixture is incubated at 55°C for 15 minutes, followed by immediate cooling on ice. To obtain high-purity circular RNA, the product is treated with RNase R to digest linear RNA. The product is then purified using Kit A, and its concentration and quality are tested.

Linear RNA (linRNA) is produced in accordance with the manufacturer’s instructions. Briefly, the construct is inserted into the pET-28a (+) plasmid and subsequently transformed into E. coli. The linRNA vector fragment is recovered via agarose gel electrophoresis. Following the manufacturer’s protocol, linRNA is transcribed using the Hiscribe T7 mRNA kit with Clean Cap Reagent AG (NEB). To enhance the purification of linRNA, unmodified linear mRNA is first treated with DNase I using the Ambion MEGAclear Transcription Cleanup Kit to remove any residual DNA. The RNA is then subjected to thermal treatment by heating to 70°C for 5 minutes, followed by rapid cooling on ice for 3 minutes. After cooling, RNA capping is performed strictly according to the manufacturer’s guidelines using a combination of mRNA cap 2′-O-methyltransferase and vaccinia capping enzyme, both sourced from NEB. The capped linear transcript is modified by the addition of a polyadenylate tail using E.coli poly (A) polymerase (NEB), in accordance with the manufacturer’s specifications. Finally, the fully processed mRNA is purified using column purification.

### Transfection

HEK 293T or HUVEC cells were seeded at a density of 5×10^5 cells per well in a 6-well plate. Following the manufacturer’s protocol, 3 μg of RNA was transfected into the cells using TransIT-mRNA reagent (Mirus). The morphology of the transfected cells was examined 24 hours post-transfection, and these cells were subsequently used for further experiments.

### Mouse and cell lines

Male C57BL/6 mice (6–8 weeks old) were purchased from Shanghai Slac Laboratory Animal Co., Ltd. All mice were maintained under specific pathogen-free conditions in the animal facility of Mengchao Hepatobiliary Hospital of Fujian Medical University. The HEK 293T cell line was obtained from the American Type Culture Collection (ATCC). The Hepa 1–6 and HUVEC cell lines, which are maintained in our laboratory, were cultured in high-glucose DMEM (Thermo) supplemented with 10% fetal bovine serum (FBS; Excell) at 37°C and 5% CO_2_.

### Cell isolation

Healthy male C57BL/6 mice were disinfected by soaking in ethanol for 15 minutes. Subsequently, the spleen was excised to collect splenocytes. T cells were isolated using Ficoll (TBD) for further experiments or cryopreservation.

### Western blot

Cells were lysed using RIPA Lysis Buffer supplemented with protease inhibitors (Roche, Indianapolis, IN) (Beyotime Biotechnology, Shanghai, China). The cells were lysed on ice for 30 minutes. Subsequently, the lysates were centrifuged at 12,000 rpm for 10 minutes to obtain the supernatant, which was used for BCA quantitative analysis. Equal amounts of protein lysates were separated by SDS-PAGE and transferred to a nitrocellulose membrane. The membrane was incubated with the following primary antibodies: anti-actin (1:2000, abcam, ab49900), anti-E-cadherin (1:2000, CST, 14472S), anti-slug (1:1000, Biorbyt), anti-snail (1:2000, CST, #3879), anti-vimentin (1:2000, CST, 5741s), anti-ZO-1 (1:2000, CST, 8193P), and anti-VEGFR (1:1000, proteintech, 13687-1-AP). Detection was performed using enhanced chemiluminescence (ECL; Millipore, Billerica, MA, USA), and the blots were imaged using the ChemiDoc MP Imaging System (Bio-Rad, Hercules, CA, USA).

### Transwell assay

Migration analysis was performed using the Transwell inserts (8-μm pores, Corning, NY, USA). For the invasion assay, the upper chamber was pre-coated with Matrigel (Corning, NY, USA). A total of 1×10^4 cells were digested, counted, and resuspended in 200 μL of DMEM, which was then added to the upper chamber. The lower chamber was filled with 500 μL of DMEM containing 10% fetal bovine serum (FBS). After 24 hours of incubation, cells that had migrated to the bottom of the upper chamber were fixed with 4% paraformaldehyde and stained with 0.5% crystal violet (Beyotime Biotechnology, Shanghai, China). The cells were then observed under a microscope. Cell counting was performed at 200× magnification, and the results from five random fields were used for statistical analysis. The experiment was repeated three times.

### Wound healing assay

Cells were digested and counted, and a total of 70 μL of the cell suspension (1×10^6^ cells/mL) was seeded on both sides of the culture inserts (Ibidi, Munich, Germany). After 12 hours of incubation, the inserts were removed. The cells were washed three times with PBS to remove debris. The initial wound distance was measured under a microscope. Wound closure was assessed every 12 hours.

### EdU staining experiment

Cells were digested, counted, and plated at a density of 1×10^5 cells per dish in Nunc glass bottom dishes (Biosharp, Anhui, China). The cells were cultured until they adhered to the surface. The EdU reagent was then diluted according to the manufacturer’s instructions (EdU kit, Beyotime, Shanghai, China) and added to the cell culture medium to co-incubate with the cells for 2 hours. The culture medium was removed, and the cells were washed three times with PBS. The cells were fixed with 4% formaldehyde at room temperature for 15 minutes. The fixative was removed, and the cells were washed three times with PBS. Subsequently, 0.3% Triton X-100 (Sigma, Germany) was added, and the cells were incubated at room temperature for 10 minutes. The cells were then washed three times with PBS, and the click reaction solution was prepared according to the manufacturer’s instructions (EdU kit). The cells were incubated with the click reaction solution at room temperature in the dark for 30 minutes. The cells were washed three times with the washing solution, and Hoechst (Beyotime, Shanghai, China) was added to stain the nuclei. The cells were incubated at room temperature in the dark for 10 minutes, washed three times with PBS, and then observed under confocal microscopy.

### Real-time cellular analysis using the maestro Z system

To initiate the real-time cellular analysis, 8 mL of phosphate-buffered saline (PBS) was added around the 96-well plate (Axion BioSystems, Shanghai, China) to maintain a humidified environment and prevent evaporation. Subsequently, 100 μL of the transfected, digested, and counted cell culture medium was carefully added to each well. The plate was then placed into the Maestro Z detection chamber. The Maestro Z software was launched, and the “Experiment Name” field was located to input the designated name of the experiment. The “Measure Baseline” function was activated to record the baseline impedance of the blank medium for 2–3 minutes. The “Settings” option in the software was selected to configure the temperature at 37°C and the CO_2_ concentration at 5%. The cell concentration was adjusted to 1×10^6^ cells/mL. A volume of 100 μL of the adjusted cell suspension was added to the corresponding wells. The plate was allowed to stand for 1 hour to facilitate cell attachment. After the cell attachment period, the seeded cell plate was placed back into the detection chamber, and the chamber door was closed. The Maestro Z system automatically recorded the cell impedance values at the set intervals. The platemap was edited according to the experimental grouping to ensure accurate data tracking. Upon completion of the experiment, the “File” menu was accessed, and “Export Experiment” was selected in the software to export and save the data for subsequent analysis.

### Quantitative real-time PCR

HCC tissues were obtained from patients who underwent curative surgery at Mengchao Hepatobiliary Hospital of Fujian Medical University. Sample collection and use were approved by Medical Ethics Committee of Mengchao Hepatobiliary Hospital of Fujian Medical University. Meanwhile, informed consent was provided by the patients. Total RNA was extracted from cells and tissues using the Trizol total RNA isolation reagent (TransGen Biotech). cDNA synthesis was performed using the Transcriptor First Strand cDNA Synthesis Kit (Roche, Basel, Switzerland). Quantitative PCR was conducted using the SYBR Green qPCR Master Mix (DBI, Ludwigshafen, Germany), with mouse U6 rRNA serving as an endogenous control. The PCR amplification protocol consisted of an initial denaturation at 95°C for 5 minutes, followed by 40 cycles of 95°C for 10 seconds and 60°C for 30 seconds. The sequences of all qPCR primers are listed in **Supplementary Table S1**.

### Vascular formation experiment

Cells were digested, counted, and seeded into 6-well plates. They were cultured until the cell confluence reached approximately 80%. The complete culture medium was then replaced with serum-free medium containing 0.2% fetal bovine serum (FBS) for a 24-hour starvation treatment. Subsequently, the cells were digested, centrifuged, and recounted. They were then resuspended in medium containing 10% FBS to achieve a single-cell suspension with a concentration of 2×10^6 cells/mL. In parallel, the matrix gel was thawed and diluted. A volume of 300 μL of the matrix gel was evenly spread into each well of a 24-well plate. The plate was then incubated in a cell incubator for 30 minutes to allow the matrix gel to solidify. Next, 100 μL of the single-cell suspension was added to each well of the 24-well plate containing the solidified matrix gel. The plate was incubated at 37°C and 5% CO_2_. Photomicrographs were taken at time points of 2 hours, 4 hours, 6 hours, and 8 hours to observe the vascular formation process.

### Encapsulation of circRNA by LNP

To encapsulate circRNA, we utilized a commercial lipid-based reagent (*in vivo*-jetRNA, Polyplus, France). Prior to use, the *in vivo*-jetRNA reagent was vortexed for 5s and centrifuged. The diluted mRNA was added to the *in vivo*-jetRNA reagent at a 1:1 ratio (1 µg of mRNA per 1 µL of *in vivo*-jetRNA reagent) and mixed uniformly by gently pipetting up and down. The resulting solution was then incubated at room temperature for 15 minutes before being administered via animal injection.

### Mouse tumor models

The animal studies were approved by the Animal Ethics Committee of Fujian Medical University Mengchao Hepatobiliary Hospital (MCHH-AEC-2024-25). For the subcutaneous tumor model, C57BL/6 mice were injected subcutaneously with 3×10^6 Hepa 1–6 cells mixed with Matrigel (ABW, Shanghai, China) on day -10. When the tumor volume reached approximately 100 mm³, the mice were randomly assigned to five groups. Immunotherapy was administered via tail vein injection on day 0 and day 7 according to the experimental groups. Tumor volume was measured every 3 days using the formula: V=length×width^2/2 (mm^3^). Mouse body weight was also recorded. When the tumor volume reached approximately 1000 mm³, the mice were euthanized. Peripheral blood was collected for biochemical analysis, and mouse tissues were harvested for hematoxylin and eosin (H&E) staining to assess the biosafety of circRNA lipid nanoparticles (LNPs). Specifically, mouse tissues were collected, fixed, paraffin-embedded, sectioned, and stained with H&E (Solarbio, Beijing, China). Tumor tissues were sent to Sevier for immunofluorescence staining to detect the immune infiltration of CD8+ and CD4+ T cells among different treatment groups.

For the *in situ* tumor model, C57BL/6 mice were injected *in situ* with 3×10^5 Hepa 1–6 cells in 20 μL on day -10. After 10 days, the size of the *in situ* liver tumor was detected using a IVIS *In Vivo* Imaging System (Perkin Elmer), and the mice were randomly divided into three groups. Immunotherapy was administered via tail vein injection on day 0 and day 7 according to the experimental groups. *In vivo* imaging was performed every 10 days to monitor tumor growth. After 60 days, the mice were euthanized. Dendritic cells (DCs) were isolated from the bone marrow for assessment of maturation by flow cytometry, and T cells were collected from the spleen for assessment of antigen-specific T cells by flow cytometry and enzyme-linked immunospot (ELISPOT) assays. Tumor tissues were harvested, fixed, paraffin-embedded, sectioned, and sent to Servicebio for immunofluorescence staining to detect the immune infiltration of CD8^+^ and CD4^+^ T cells among different treatment groups.

### Statistical analysis

Statistical analyses were conducted using GraphPad Prism (version 8.0). For normally distributed variables, comparisons between two groups were performed using the Student’s t-test. For data with non-normal distributions, the Mann-Whitney U test was employed to assess statistical significance. All data were presented as mean ± SD from at least three independent experiments with duplicate or triplicate samples. p < 0.05 was considered a statistically significant difference in the analysis.

## Data Availability

The raw data supporting the conclusions of this article will be made available by the authors, without undue reservation.

## References

[1] Song R Ma S Xu J Ren X Guo P Liu H. A novel polypeptide encoded by the circular RNA ZKSCAN1 suppresses HCC via degradation of mTOR. Mol Cancer. (2023) 22:16. doi: 10.1186/s12943-023-01719-9 36691031 PMC9869513

[2] Wu X Xiao S Zhang M Yang L Zhong J Li B. A novel protein encoded by circular SMO RNA is essential for Hedgehog signaling activation and glioblastoma tumorigenicity. Genome Biol. (2021) 22:33. doi: 10.1186/s13059-020-02250-6 33446260 PMC7807754

[3] Wesselhoeft RA Kowalski PS Anderson DG. Engineering circular RNA for potent and stable translation in eukaryotic cells. Nat Commun. (2018) 9:2629. doi: 10.1038/s41467-018-05096-6 29980667 PMC6035260

[4] Chen R Wang SK Belk JA Amaya L Li Z Cardenas A. Engineering circular RNA for enhanced protein production. Nat Biotechnol. (2023) 41:262–72. doi: 10.1038/s41587-022-01393-0 PMC9931579 35851375

[5] Wang F Cai G Wang Y Zhuang Q Cai Z Li Y. Circular RNA-based neoantigen vaccine for hepatocellular carcinoma immunotherapy. MedComm (2020). (2024) 5:e667. doi: 10.1002/mco2.v5.8 39081513 PMC11286538

[6] Li X-S Xia F. Immunotherapy for hepatocellular carcinoma: molecular pathogenesis and clinical research progress. Oncol Trans Med. (2023) 9:206–12. doi: 10.1097/ot9.0000000000000013

[7] Rafiq S Yeku OO Jackson HJ Purdon TJ van Leeuwen DG Drakes DJ. Targeted delivery of a PD-1-blocking scFv by CAR-T cells enhances anti-tumor efficacy *in vivo* . Nat Biotechnol. (2018) 36:847–56. doi: 10.1038/nbt.4195 PMC6126939 30102295

[8] Rani V Sengar RS. Biogenesis and mechanisms of microRNA-mediated gene regulation. Biotechnol Bioeng. (2022) 119:685–92. doi: 10.1002/bit.v119.3 34979040

[9] Wang C Wang X Su Z Fei H Liu X Pan Q. MiR-25 promotes hepatocellular carcinoma cell growth, migration and invasion by inhibiting RhoGDI1. Oncotarget. (2015) 6:36231–44. doi: 10.18632/oncotarget.v6i34 PMC4742173 26460549

[10] El-Mezayen H Yamamura K Yusa T Nakao Y Uemura N Kitamura F. MicroRNA-25 exerts an oncogenic function by regulating the ubiquitin ligase fbxw7 in hepatocellular carcinoma. Ann Surg Oncol. (2021) 28:7973–82. doi: 10.1245/s10434-021-09778-2 33886022

[11] Zeng Z Li Y Pan Y Lan X Song F Sun J. Cancer-derived exosomal miR-25-3p promotes pre-metastatic niche formation by inducing vascular permeability and angiogenesis. Nat Commun. (2018) 9:5395. doi: 10.1038/s41467-018-07810-w 30568162 PMC6300604

[12] Liu X Abraham JM Cheng Y Wang Z Wang Z Zhang G. Synthetic circular RNA functions as a miR-21 sponge to suppress gastric carcinoma cell proliferation. Mol Ther Nucleic Acids. (2018) 13:312–21. doi: 10.1016/j.omtn.2018.09.010 PMC6197335 30326427

[13] Rupaimoole R Slack FJ. MicroRNA therapeutics: towards a new era for the management of cancer and other diseases. Nat Rev Drug Discov. (2017) 16:203–22. doi: 10.1038/nrd.2016.246 28209991

[14] Gregory PA Bert AG Paterson EL Barry SC Tsykin A Farshid G. The miR-200 family and miR-205 regulate epithelial to mesenchymal transition by targeting ZEB1 and SIP1. Nat Cell Biol. (2008) 10:593–601. doi: 10.1038/ncb1722 18376396

[15] Korpal M Lee ES Hu G Kang Y. The miR-200 family inhibits epithelial-mesenchymal transition and cancer cell migration by direct targeting of E-cadherin transcriptional repressors ZEB1 and ZEB2. J Biol Chem. (2008) 283:14910–4. doi: 10.1074/jbc.C800074200 PMC3258899 18411277

[16] Park SM Gaur AB Lengyel E Peter ME. The miR-200 family determines the epithelial phenotype of cancer cells by targeting the E-cadherin repressors ZEB1 and ZEB2. Genes Dev. (2008) 22:894–907. doi: 10.1101/gad.1640608 18381893 PMC2279201

[17] Medina PP Nolde M Slack FJ. OncomiR addiction in an *in vivo* model of microRNA-21-induced pre-B-cell lymphoma. Nature. (2010) 467:86–90. doi: 10.1038/nature09284 20693987

[18] Liangqi C Xuewei Y Yubin C Dawei Z Xiao F Jiang P. Exosomal miR-21 regulates the TETs/PTENp1/PTEN pathway to promote hepatocellular carcinoma growth. Mol Cancer. (2019) 18:148. doi: 10.1186/s12943-019-1075-2 31656200 PMC6815431

[19] Arghiani N Matin MM. miR-21: A key small molecule with great effects in combination cancer therapy. Nucleic Acid Ther. (2021) 31:271–83. doi: 10.1089/nat.2020.0914 33891511

[20] Hong DS Kang YK Borad M Sachdev J Ejadi S Lim HY. Phase 1 study of MRX34, a liposomal miR-34a mimic, in patients with advanced solid tumours. Br J Cancer. (2020) 122:1630–7. doi: 10.1038/s41416-020-0802-1 PMC7251107 32238921

[21] van Zandwijk N Pavlakis N Kao SC Linton A Boyer MJ Clarke S. Safety and activity of microRNA-loaded minicells in patients with recurrent Malignant pleural mesothelioma: a first-in-man, phase 1, open-label, dose-escalation study. Lancet Oncol. (2017) 18:1386–96. doi: 10.1016/S1470-2045(17)30621-6 28870611

[22] Chen L Zhang K Shi Z Zhang A Jia Z Wang G. A lentivirus-mediated miR-23b sponge diminishes the Malignant phenotype of glioma cells *in vitro* and *in vivo* . Oncol Rep. (2014) 31:1573–80. doi: 10.3892/or.2014.3012 24503899

[23] Peng Y Croce CM. The role of MicroRNAs in human cancer. Signal Transduct Target Ther. (2016) 1:15004. doi: 10.1038/sigtrans.2015.4 29263891 PMC5661652

[24] Su ZX Zhao J Rong ZH Geng WM Wu YG Qin CK. Upregulation of microRNA-25 associates with prognosis in hepatocellular carcinoma. Diagn Pathol. (2014) 9:47. doi: 10.1186/1746-1596-9-47 24593846 PMC4016611

[25] Zhai D Zhou Y Kuang X Shao F Zhen T Lin Y. Lnc NR2F1-AS1 promotes breast cancer metastasis by targeting the miR-25-3p/ZEB2 axis. Int J Med Sci. (2023) 20:1152–62. doi: 10.7150/ijms.86969 PMC10416723 37575267

[26] Wan W Wan W Long Y Li Q Jin X Wan G. MiR-25-3p promotes Malignant phenotypes of retinoblastoma by regulating PTEN/Akt pathway. BioMed Pharmacother. (2019) 118:109111. doi: 10.1016/j.biopha.2019.109111 31336343

[27] Ren T Liu C Hou J Shan F. Hsa_circ_0043265 suppresses proliferation, metastasis, EMT and promotes apoptosis in non-small cell lung cancer through miR-25-3p/FOXP2 pathway. Onco Targets Ther. (2020) 13:3867–80. doi: 10.2147/OTT.S235231 PMC7213897 32440153

[28] Ghaderi SS Riazi-Rad F Qamsari ES Bagheri S Rahimi-Jamnani F Sharifzadeh Z. Development of a human phage display-derived anti-PD-1 scFv antibody: an attractive tool for immune checkpoint therapy. BMC Biotechnol. (2022) 22:22. doi: 10.1186/s12896-022-00752-8 35996120 PMC9396865

[29] Chen J Zhu T Jiang G Zeng Q Li Z Huang X. Target delivery of a PD-1-TREM2 scFv by CAR-T cells enhances anti-tumor efficacy in colorectal cancer. Mol Cancer. (2023) 22:131. doi: 10.1186/s12943-023-01830-x 37563723 PMC10413520

[30] Ju F Luo Y Lin C Jia X Xu Z Tian R. Oncolytic virus expressing PD-1 inhibitors activates a collaborative intratumoral immune response to control tumor and synergizes with CTLA-4 or TIM-3 blockade. J Immunother Cancer. (2022) 10:e004762. doi: 10.1136/jitc-2022-004762 35688558 PMC9189843

