# Supplementary figures and images for: Utilization of artificial circular RNAs as miRNA sponges and anti-PD-1 scFv expression platforms to suppress hepatocellular carcinoma progression
Source: Front Immunol. 2025 Jun 11;16:1609165. doi: 10.3389/fimmu.2025.1609165 (PMC12187678; doi:10.3389/fimmu.2025.1609165)

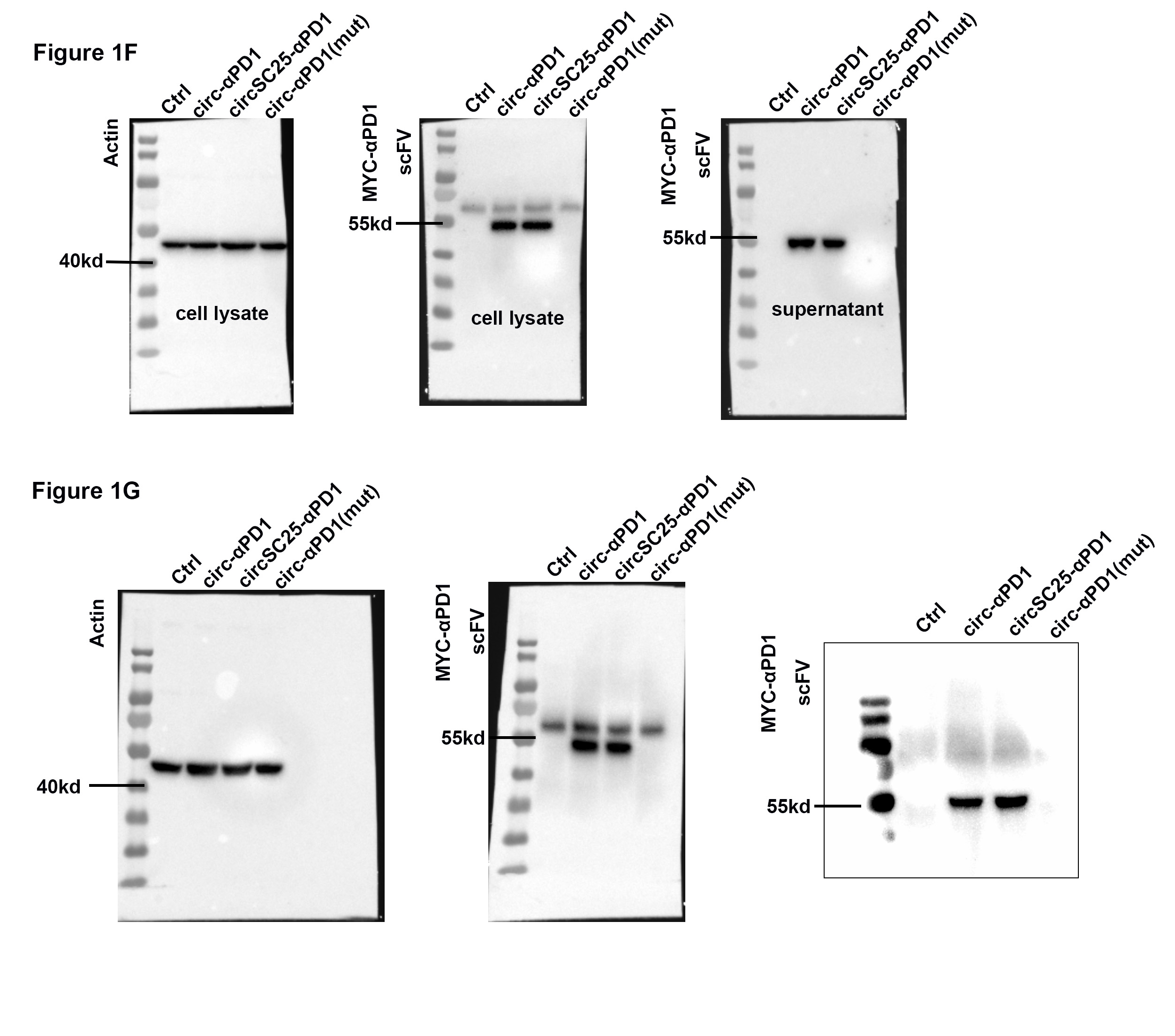

Supplement: Supplementary file 1 [file DataSheet1.zip › raw data 1/Figure 1F,1H western blot full scan.jpg]

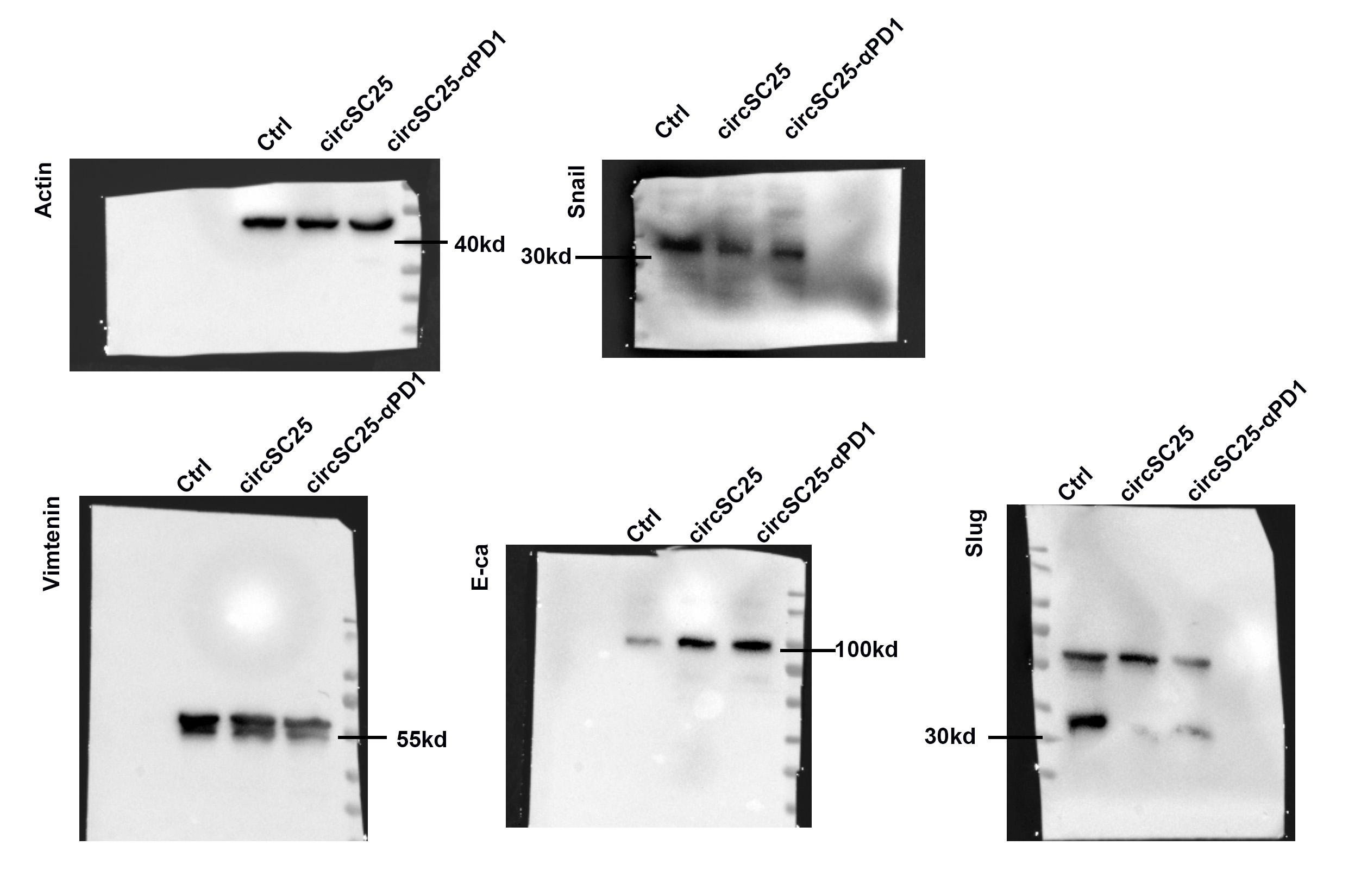

Supplement: Supplementary file 1 [file DataSheet1.zip › raw data 1/Figure 2E western blot full scan.jpg]

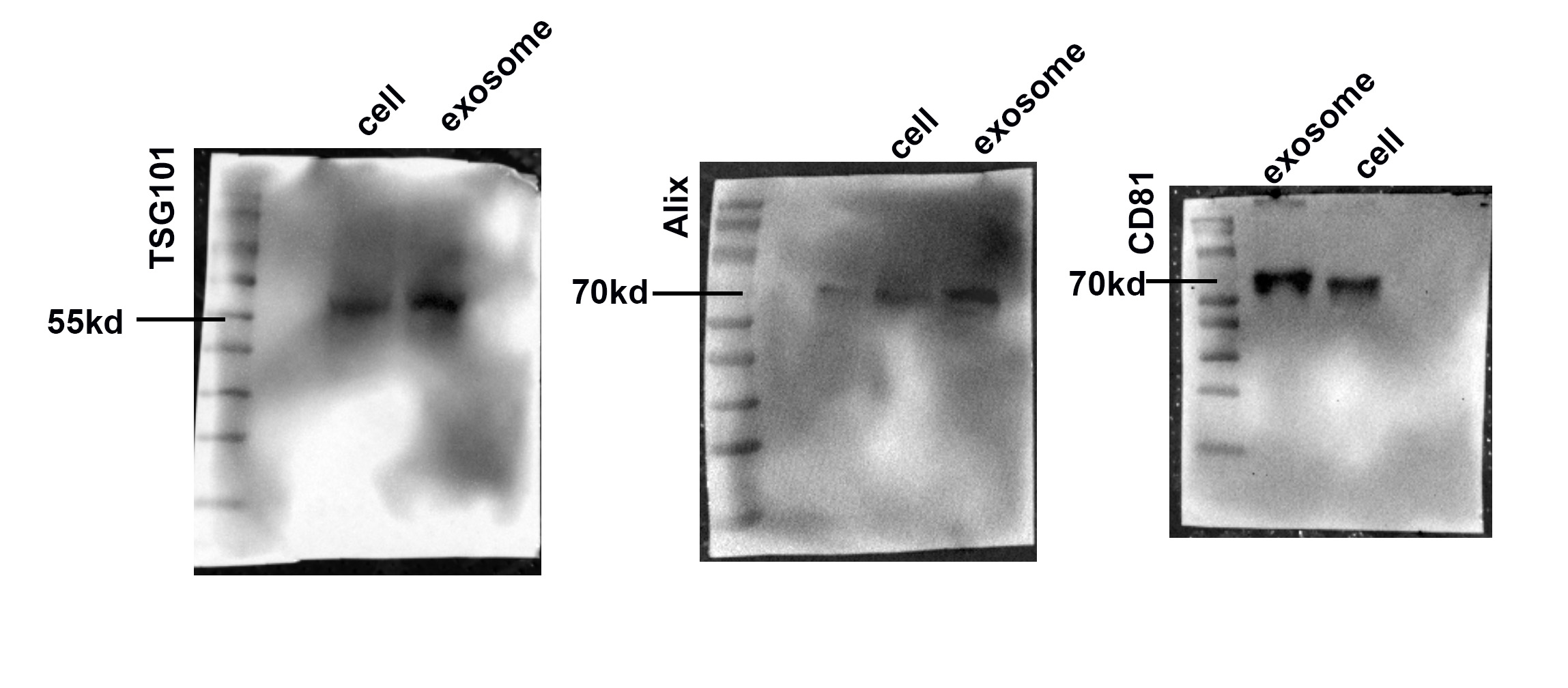

Supplement: Supplementary file 1 [file DataSheet1.zip › raw data 1/Figure 3A western blot full scan.jpg]

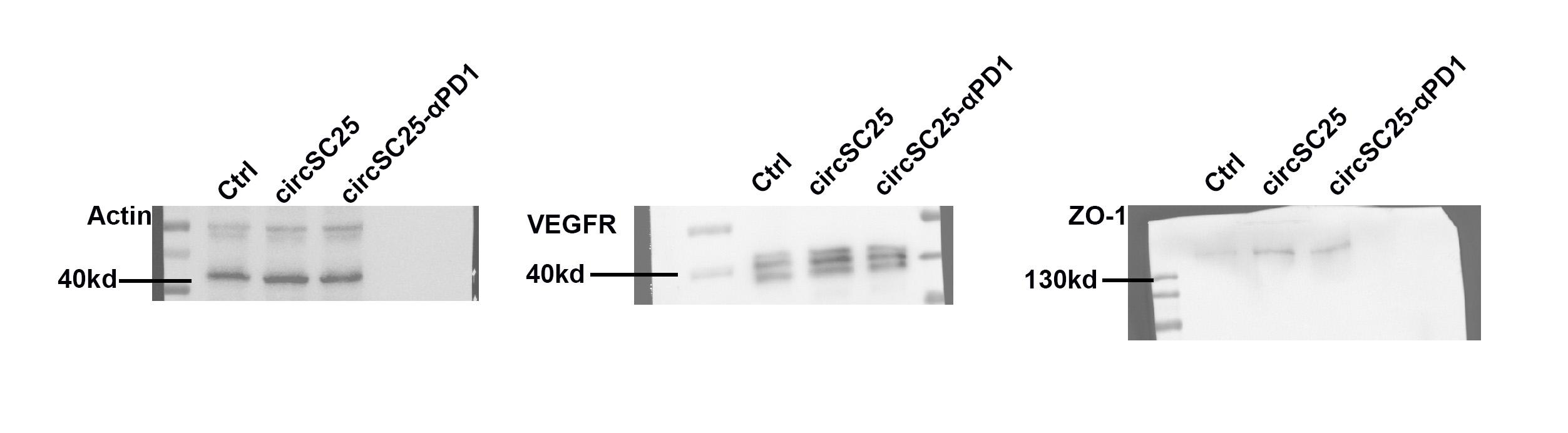

Supplement: Supplementary file 1 [file DataSheet1.zip › raw data 1/Figure 3E western blot full scan.jpg]
