# Supplementary material for: Utilization of artificial circular RNAs as miRNA sponges and anti-PD-1 scFv expression platforms to suppress hepatocellular carcinoma progression
Source: Front Immunol. 2025 Jun 11;16:1609165. doi: 10.3389/fimmu.2025.1609165 (PMC12187678; doi:10.3389/fimmu.2025.1609165)
Supplement: Supplementary file 3 [file Table1.docx]

**Supplementary Table S1. qPCR primer sequences**

| qPCR primer sequences | |
| --- | --- |
| Gene symbol | Sequences (from 5’ to 3’) |
| Mmu-miR-25-3p | F: GCGCATTGCACTTGTCTCG |
|  | R: AGTGCAGGGTCCGAGGTATT |
| Mmu-miR-25-5p | F: GAGGCGGAGACTTGGGC |
|  | R:AGTGCAGGGTCCGAGGTATT |
| Mmu-U6 | F: CTCGCTTCGGCAGCACATATACT |
|  | R: ACGCTTCACGAATTTGCGTC |
| homo-miR-25 | F:GGCCAGTGTTGAGAGGC |
|  | R:GGCCGGCACTGTCAGACCG |
| 18s | F: CACCAGACTTGCCCTCCA |
|  | R:AGAAACGGCTACCACATCCA |
